# Associations of Sex and Sport Contact-Level with Recovery Timelines Among Collegiate Athletes with Sport-Related Concussion

**DOI:** 10.1186/s40798-024-00742-4

**Published:** 2024-07-29

**Authors:** Bernadette A. D’Alonzo, Andrea L. C. Schneider, Ian J. Barnett, Christina L. Master, Abigail C. Bretzin, Douglas J. Wiebe, Ivy League-Big Ten Epidemiology of Concussion Study Investigators, Beth Conroy, Thomas Bottiglieri, Amy Sucheski-Drake, Kathryn J. Harris, Kristine A. Karlson, Jonathan D. Lichtenstein, Arun J. Ramappa, Randy Ballard, Nicholas L. Port, Andrew R. Peterson, Bradley D. Hatfield, Mathew R. Saffarian, James T. Eckner, Erin Moore, Suzanne Hecht, Cary R. Savage, Kate Higgins, Matthew J. Nerrie, Anthony Erz, Brian J. Sennett, Michael Gay, Sasha Steinlight, Scott Lawrance, Jason Womack, Carrie Esopenko, Elizabeth C. Gardner

**Affiliations:** 1 Department of Biostatistics, Epidemiology and Informatics, Perelman School of Medicine, University of Pennsylvania, Philadelphia, PA USA; 2 Department of Neurology, Perelman School of Medicine, University of Pennsylvania, Philadelphia, PA USA; 3 Department of Pediatrics, Perelman School of Medicine, University of Pennsylvania, Philadelphia, PA USA; 4 Center for Injury Research and Prevention, Children’s Hospital of Philadelphia, Philadelphia, PA USA; 5 Sports Medicine and Performance Center, Children’s Hospital of Philadelphia, Philadelphia, PA USA; 6 Injury Prevention Center, Department of Emergency Medicine, University of Michigan, Ann Arbor, MI USA

**Keywords:** Athletes, Sport concussion, Traumatic brain injury, Recovery

## Abstract

**Background:**

Growing interest has motivated recent studies to examine differences in recovery after sport-related concussion (SRC) by sex. However, heterogeneity in study design, participants, and recovery outcomes has led to mixed findings. Further work is needed to evaluate potential differences by sex and to investigate the role of related characteristics, such as sport contact-level, in recovery timelines. This study aimed to investigate whether concussion recovery trajectories differ by sex, considering a priori clinical and demographic covariates, and accounting for the sequence of recovery outcomes. Our secondary question was whether sport contact-level modifies the relationship between sex and time to outcomes. Using data from the Ivy League–Big Ten Epidemiology of Concussion Study, we included SRCs reported across five academic years; 2015–2020 (February 2020). We used Cox proportional hazards regressions to estimate associations between sex and time from injury to three outcomes: (1) symptom resolution, (2) return to academics, (3) return to full play, accounting for measured confounders.

**Results:**

Among 1160 SRCs (male, n = 667; female, n = 493) with complete data, median age overall was 20 years (25th-75th percentiles:19–21), and most occurred among athletes playing high-contact sports (78.0%). Males were slightly more likely to complete symptom resolution over time compared to females (HR = 1.18, 95%CI = 1.05–1.33), but results were attenuated in fully adjusted models (HR 1.13, 95%CI = 0.99–1.29). Similarly, the HR of full academic return for males compared to females was 1.22 (95%CI = 1.07–1.38), but was attenuated in fully adjusted models (HR = 1.11, 95%CI = 0.97–1.28). The HR of full return to play for males compared to females was 1.14 (95%CI = 1.02–1.28), and was attenuated after adjustment (HR = 1.06, 95%CI = 0.93–1.20) as well. The interaction between sex and playing a high/low-contact sport was not statistically significant across models, though differences were apparent.

**Conclusions:**

Among a cohort of collegiate athletes with SRC, recovery timelines appeared similar between male and female athletes, adjusting for measured confounders. Differences by sex, considering sport contact-level, were evident and may be important clinically and in future studies. This study used robust methods, accounting for nesting in the sequence of RTP outcomes. Results inform concussion management protocols and planned qualitative work to further elucidate how collegiate athletes experience concussion recovery.

**Key Points:**

- Heterogeneity in study design, participants, and recovery outcomes has led to mixed findings in determining differences in recovery trajectories after concussion by sex.
- We found that having longer time to symptom resolution, and also the sequence of having academic return before symptoms resolve and longer time to academic return were confounders in the relationship between sex and RTP timelines. Time to sequential recovery outcomes appeared similar between male and female athletes, adjusting for observable confounders. Further differences by sex were evident when considering contact-level, and may be important to consider clinically and in future research.
- Results indicate that differences in concussion recovery trajectories by sex may be largely attributed to and driven by differences in sports with a men’s or women’s team only, such as football, and this should be explored further.

**Supplementary Information:**

The online version contains supplementary material available at 10.1186/s40798-024-00742-4.

## Introduction

### Background

Over 1 million sport-related concussions (SRC) affect youth (age 9–22 years) annually [1]. In the collegiate setting specifically, concussions comprise roughly 6% of injuries among NCAA athletes [2]. The International Concussion in Sport Group (CISG) and National Athletic Training Association (NATA) determine the diagnostic, treatment and return-to-play (RTP) guidelines; a graduated progression to sport consisting of 24–48 h rest period followed by a 6-stage return to sport/RTP strategy [3–6]. The stages, activities, and goals of return to sport consist generally of the athlete engaging in symptom limited activity, followed by light aerobic exercise, sport-specific exercise, non-contact and then full-contact practice and training drills, and then return to normal/full sport. A graduated return to academics strategy, whereby the athlete is introduced to academic activities, and followed through part-time, and full-time return, is also implemented in parallel. However, the length and quality of recovery greatly varies between individuals following a concussion, with the majority of athletes having symptoms resolve within two weeks, and return to play within three weeks after injury [7–9]. Estimates suggest 10–20% may experience symptoms beyond two weeks, with persistent symptoms still occurring at three months, and a smaller portion, still, who may experience chronic symptoms up to a year after injury [3, 10]. These data are in contrast to findings from a recent CISG systematic review and meta-analysis of clinical recovery and time to return to school and sport after concussion where the heterogeneity of study designs, participants, and outcomes led to results suggesting that all athletes (regardless of age and sex) have similar recovery patterns [7]. Taken together, further work is needed to identify factors which may indicate individuals at risk for incomplete recovery or longer time to recovery.

Investigations into predictors of clinical recovery have identified some characteristics associated with longer clinical recovery and return-to-play timelines; including: older age, female sex, history of prior concussions, greater number and severity of symptoms, and emerging evidence of symptom type, including affective symptoms [7, 11–15]. However, these studies have reported mixed findings [7, 11], and largely been limited in terms of sample size of collegiate athletes and analysis. In terms of analysis, specifically, few quantify the impact of these factors (or combination of factors) on differences in time to specific outcomes (e.g., symptom resolution, return to academics, return to play) [6, 9, 11]. In addition, statistical models have not accounted for the stepwise progression that athletes are guided through during concussion management, which could affect conclusions made around length of return-to-play timelines, warranting further work.

The objective of this study was to determine whether concussion recovery trajectories in the collegiate athlete population differ by sex, evaluating the role of covariates, and accounting for the sequence of RTP outcomes. As prior research suggests, we expected female athletes to display longer time to symptom resolution, return to full academics, and return to full play. We also hypothesized that sport contact-level would modify the relationship between sex and time to recovery outcomes, with low-contact sport athletes exhibiting longer recovery timelines.

## Methods

### Study Design and Study Population

We used data from the Ivy League–Big Ten Epidemiology of Concussion Study (Ivy–B1G Study)—a prospective, multisite, observational study of student-athlete concussions across 20 Ivy League and Big Ten universities. Previous publications outline the methods of the Ivy-B1G Study in detail and describe the overall cohort [8]. The University of Pennsylvania institutional review board serves as the central institutional review board for this study. All participants provided informed consent. This study follows the Strengthening the Reporting of Observational Studies in Epidemiology (STROBE) reporting guidelines for cohort studies.

Of note, in the present study, we aim to be mindful concerning the appropriate use of the terms sex and gender; whereas sex is based on biological or physiological attributes, and gender refers to a social construct, and both characteristics affect health [16]. Here, we present our results referring to sex (“male; female”) and also using the terms ‘‘women; men’’ and ‘‘athletes on men’s/women’s teams’’ throughout to reflect how data were collected in the Ivy–B1G Study during the study period [8].

For the present analysis, we included SRCs reported in varsity sports across five consecutive academic years; 2015–16 through 2019–20 (February 2020), as all variables of interest were collected during these years. Of 1904 total concussions, we excluded 739 for missing outcome data, and 5 for missing covariate data, resulting in 1160 complete records included in analyses (Appendix Figure 1).

### Demographic and Clinical Characteristics

The main exposure of interest is self-reported sex (male; female). Class year is defined as the academic year in school (freshman, sophomore, junior, senior, fifth year), and age is reported as the student-athlete’s age at the time of the concussion. 27 sports are represented in the Ivy-B1G study and we categorized them into high/low contact sport type, consistent with previous work [9]. Symptom count is defined as the total number of unique symptoms from the 22-item SCAT symptom evaluation the athlete reported throughout their concussion recovery. Concussion history is defined as the number of previous concussions at the time of injury. Academic accommodations (yes/no) is defined as whether the athlete received academic accommodations during their concussion recovery.

### Recovery Outcomes

Time to the outcome symptom resolution was defined as the number of days between the date of concussion injury and the date when the athlete self-reported being symptom-free. Time to return to academics was defined as the number of days between concussion injury and the first day the athlete returned to full academic coursework, in class [6]. Time to return to full play was defined as the number of days between concussion injury and the date the athlete was medically cleared by a physician within the sports medicine or athletics department to return to full athletic participation [9]. Both time to return to academics and full play are based on determinations made by athletic trainers and team physicians. As previously described [6], each of these recovery outcomes is relevant to one of the stages in the return-to-sport and return-to-academics strategies in the 2017 CISG consensus statement and 2014 NATA position statement on concussion [3, 5].

### Statistical Analysis

We summarize participant characteristics overall and stratified by sex as median and 25th–75th percentiles for continuous variables and as counts and proportions for categorical variables. We test for between-sex differences among baseline covariates using Wilcoxon rank-sum and chi-square tests for categorical variables.

In our survival analyses, we separately estimate associations between sex and three outcomes: (1) symptom resolution, (2) return to academics, (3) return to full play. Student athletes were followed from the time of concussion to the outcome of interest, loss-to-follow-up, or administrative censoring on June 1 of the academic year [17]. Academic year was chosen since athletic training care, and therefore surveillance for the study, is less consistent and reliable over the summer months [8]. We present Kaplan–Meier (KM) curves with log-rank tests to estimate an overall difference in time from date of injury to each of the three outcomes by sex and then further stratified by high/low-contact sport.

We use Cox proportional hazards regressions to estimate hazard ratios (HR) for the association of sex with the outcomes of symptom resolution, return to academics, and return to full play. We assessed whether the proportional hazards assumption was satisfied graphically by inspecting log–log plots for evidence of crossing, and statistically with post hoc Schoenfeld residuals tests by examining for *p*-values < 0.05 globally and for each variable in the model individually, indicative of violation in proportional hazards [18–22]. We found the proportional hazards assumption was met for each model, meaning that our hazard functions were proportional over time, and so we continued with Cox proportional hazards regressions. We proceeded with our three models in three stages. First, we estimate the unadjusted association between sex and the outcome. Second, we adjusted for a priori-specified confounding variables including class year, symptom count, concussion history, and academic accommodations. In building our models, in order to account for the nesting of RTP outcomes, we included variables representing the other (preceding) outcome(s) as covariates, such as whether the athlete’s time to symptom resolution was greater than the median number of days, and whether the athlete experienced academic return before symptoms resolved. To determine which covariates/confounders led to the greatest attenuation in the relationship between sex and each outcome, we loaded each covariate into the model with sex and examined the HR effect estimate, 95% CI, and standard errors. Third, we tested for effect modification by contact-level using the Wald test on the regression coefficient estimate for the interaction between sex and contact-level. We then stratified each adjusted model by level of contact (high/low-contact sport).

We conducted two sensitivity analyses to investigate and account for potential differences by school/study site and sport. The first was a multilevel survival model to account for potential clustering by school/study site, where we set the strata to be school. Here, we were testing the assumption that all schools follow the same CISG/NATA protocols; i.e., the concussion management is the same across study sites. The second was a subgroup analysis restricting the study population to include only those individuals playing high-contact sports with comparable rules (basketball, soccer, rugby, and water polo) between men’s/women’s teams (n = 325; 138 male, 187 female). We performed this analysis to determine whether a difference in time to outcomes by sex could be attributed to a greater proportion of male athletes in the study playing football, sprint football, and wrestling, as sports played only by male athletes with high incidence of concussion.

We also performed two sensitivity analyses to investigate the potential impact of missing data among 739 cases in our study with missing outcome data (notably, academic return and symptom resolution are also conceptualized as covariates in statistical models for other outcomes). In the first sensitivity analysis, we used a conservative approach, and made the assumption that if a case had a return to full play date, but date of symptom resolution and/or date of return to full academics was missing, then these outcome(s) occurred on the same date as return to full play. Conversely, if a return to full play date was missing, we assumed that they never returned to full play, and made their return to full play date equal to the censoring date (June 1). In the second sensitivity analysis, we used a data-driven approach. We assumed that missingness was based on what we observed in our complete case cohort. If a case had a return to full play date, but date of symptom resolution and/or date of return to full academics was missing, we used the median number of days between date of symptom resolution and full play, and also from return to full academics and full play from the complete case cohort to generate a new date(s) for symptom resolution and/or full academics. Conversely, if a case was missing a date of return to full play, we used the median number of days between symptom resolution and full play from the complete case data to generate a date of return to full play.

A 2-sided *p* < 0.05 was set as the threshold of significance for each hypothesis test. Statistical analyses were performed using StataBE 17.1 (StataCorp).

## Results

### Description of Population

A total of 1160 concussions (male, n = 667; female, n = 493) occurred between 2015 and 2020 across participating sites (Table 1). The median age at time of concussion overall was 20 years (25th–75th percentiles: 19–21). The majority of concussions occurred among athletes playing a high-contact sport (78.0%), but this was different by sex, with fewer females (67.5%) playing a high-contact sport than males (90.6%). Median symptom count (SCAT symptom evaluation) was 11 (25th–75th percentiles: 7–15) for both males and females. For more than one-half of student-athletes, this was their first concussion documented, including prior to collegiate play (54.4%). Roughly one-half of student-athletes received academic accommodations (49.8%) after their concussion.

**Table 1:** Characteristics of SRC in Ivy-B1G Study athletes, complete case (2015–16 to 2019–20)

Characteristic, n(%)	Overall	Female	Male	*p*-value
Total	1160	493 (42.5)	667 (57.5)	
**Demographic**				
Class year				0.24
Freshman	309 (26.6)	146 (29.6)	163 (24.4)	
Sophomore	353 (30.4)	148 (30.0)	205 (30.7)	
Junior	281 (24.2)	118 (23.9)	163 (24.4)	
Senior	204 (17.6)	77 (15.6)	127 (19.0)	
Fifth year	13 (1.1)	4 (0.6)	9 (1.3)	
Age, median (25th–75th percentiles)*	20 (19–21)	19 (19–20)	20 (19–21)	< 0.001
High-contact sport**	905 (78.0)	301 (67.5)	604 (90.6)	< 0.001
**Clinical**				
Symptom count, median (25th-75th percentiles)	11 (7–15)	11 (7–15)	11 (6–15)	0.14
Concussion history				0.97
0	631 (54.4)	270 (54.8)	361 (54.1)	
1	319 (27.5)	135 (27.4)	184 (27.6)	
≥ 2	210 (18.1)	88 (17.8)	122 (18.3)	
Academic accommodations	578 (49.8)	262 (53.1)	495 (47.4)	0.05

*Age at date of concussion, missing for n = 3 (male)

**n = 27 sports; *High-contact*: Basketball, Field hockey, Football, Ice hockey, Lacrosse, Rugby, Soccer, Sprint football, Water polo, Wrestling; *Low-contact*: Baseball, Cheerleading, Diving, Equestrian, Fencing, Golf, Gymnastics, Polo, Rowing/Crew, Sailing, Skiing, Softball, Squash, Swimming, Tennis, Track & field/Cross country, Volleyball

### Time to Recovery Outcomes

**Fig. 1:**
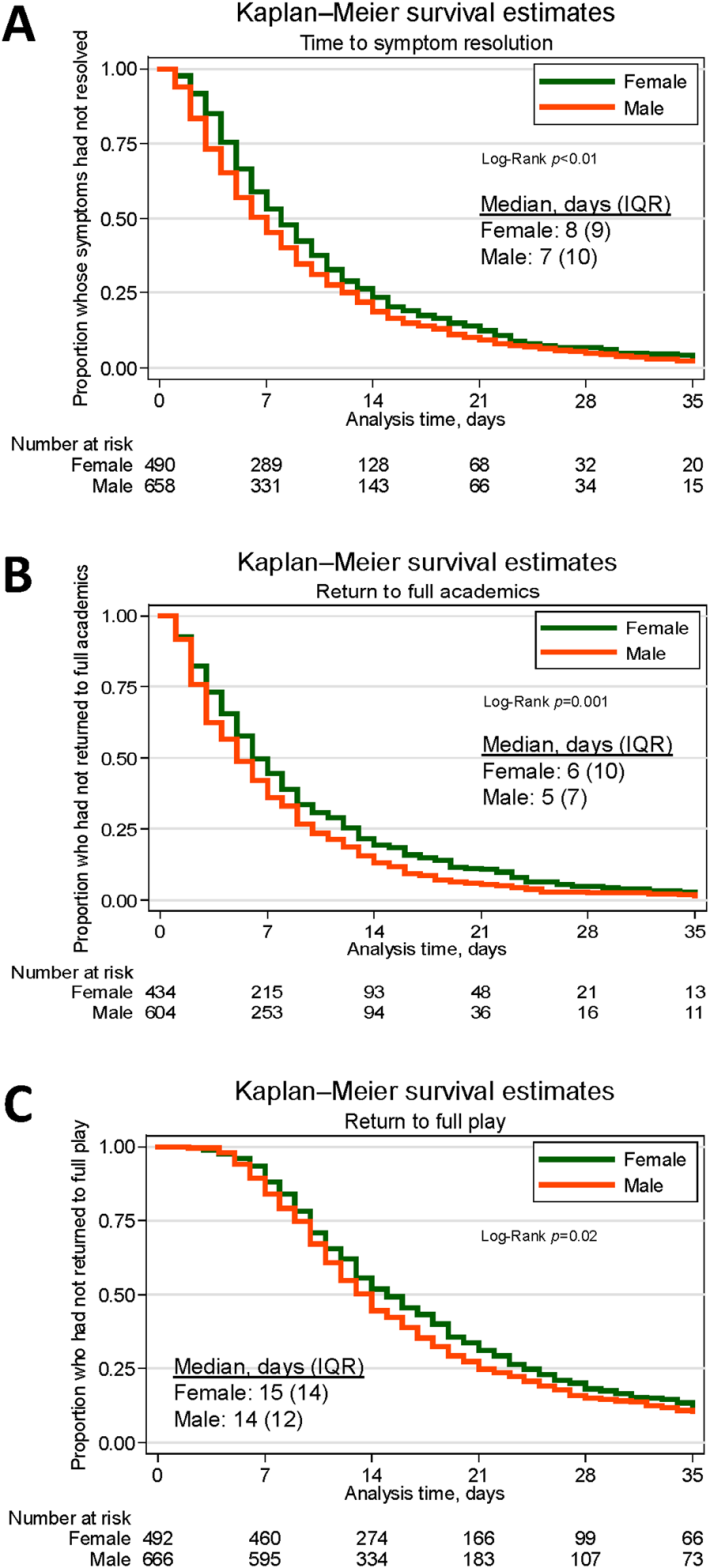
Cumulative time from injury to **A** symptom resolution, **B** academic return, **C** full play return by sex

**Fig. 2:**
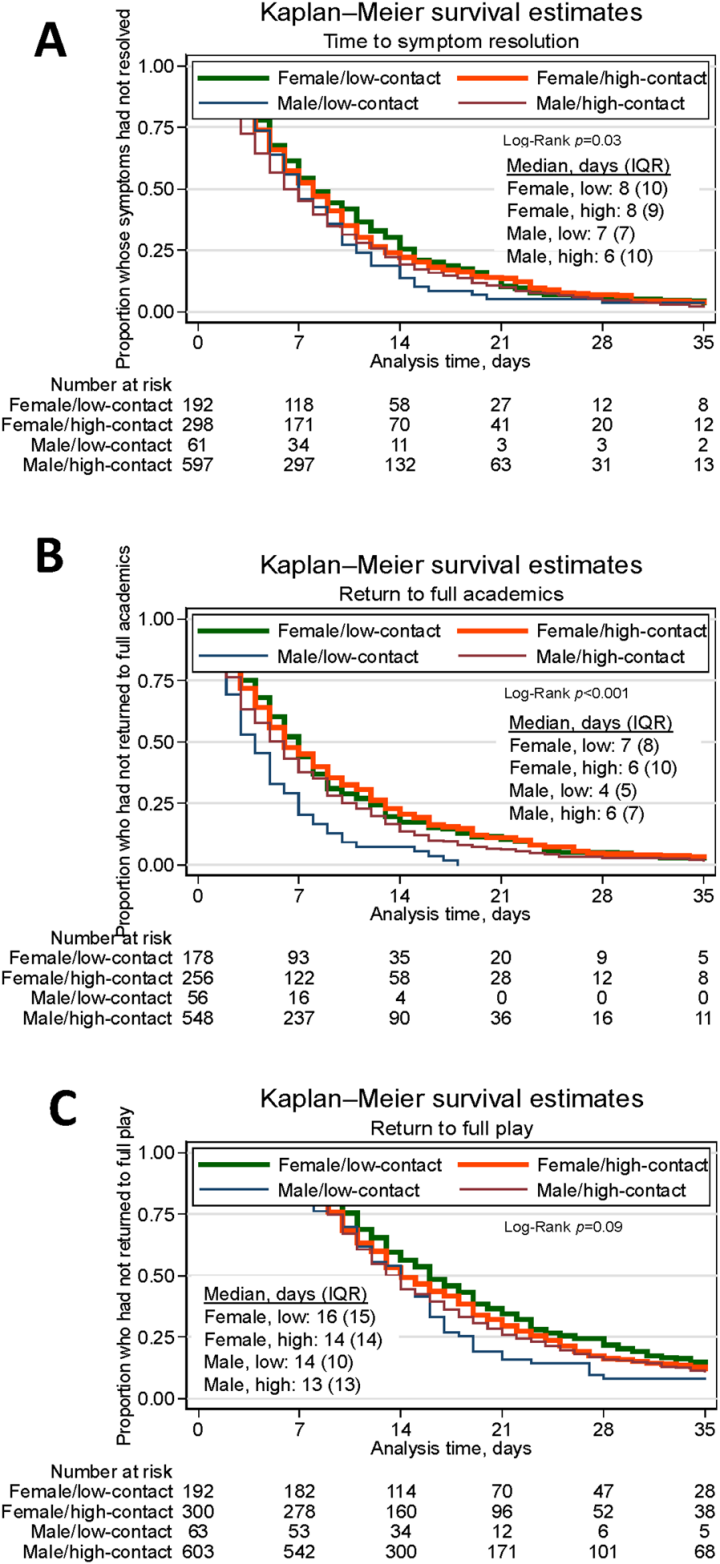
Cumulative time from injury to **A** symptom resolution, **B** academic return, **C** full play return by sex and high/low-contact level

Cumulative time to symptom resolution, full return to academics, and full return to play was consistently shorter among males compared to females (females were a median of 1 day longer than males for all outcomes) (Fig. 1). With further stratification by sex and high/low-contact-level, cumulative time to symptom resolution and cumulative time to return to full play was shortest for males playing high-contact sports, while the shortest cumulative time to return to full academics was observed for males playing low-contact sports (Fig. 2).

#### Symptom Resolution

Males were slightly more likely to have symptoms resolve over time compared to females (HR = 1.18, 95% CI 1.05–1.33) (Table 2). Overall, there was a 4.2% attenuation in the HR for sex (HR = 1.13, 95% CI 0.99–1.29) after adjusting for class year, high-contact sport, symptom count, and concussion history. The greatest attenuation in the HR estimate for sex was due to symptom count.

**Table 2:** Hazard ratios for association between sex and time to recovery outcomes

Outcome: Time from injury to symptom resolution						
	HR	95% CI	*p*-value	HR	95% CI	*p*-value
Characteristic	Unadjusted			Adjusted (n = 1148)		
Male	1.18	1.05–1.33	< 0.01	1.13	0.99–1.29	0.05
Class year						
Freshman				Ref	Ref	Ref
Sophomore				0.90	0.77–1.05	0.18
Junior				1.08	0.91–1.27	0.38
Senior				1.04	0.87–1.24	0.70
Fifth year				0.85	0.49–1.48	0.57
High-contact sport				1.07	0.92–1.24	0.41
Symptom count				0.94	0.93–0.95	< 0.001
Concussion history						
0				Ref	Ref	Ref
1				0.81	0.71–0.93	< 0.01
≥ 2				0.76	0.64–0.89	0.001

Outcome: Time from injury to return to full academics						
Characteristic	HR	95% CI	*p*-value	HR	95% CI	*p*-value
	Unadjusted			Adjusted (n = 1038)		
Male	1.22	1.07–1.38	< 0.01	1.11	0.97–1.28	0.13
Class year						
Freshman				Ref	Ref	Ref
Sophomore				0.89	0.75–1.04	0.15
Junior				0.94	0.79–1.12	0.48
Senior				0.97	0.80–1.18	0.76
Fifth year				0.75	0.41–1.37	0.34
High-contact sport				0.85	0.72–1.00	0.05
Symptom count				0.98	0.97–0.99	0.02
Concussion history						
0				Ref	Ref	Ref
1				1.01	0.87–1.17	0.89
≥ 2				0.86	0.72–1.01	0.08
Academic accommodations-yes				0.58	0.50–0.66	< 0.001
Symptom resolution > median				0.38	0.33–0.45	< 0.001

Outcome: Time from injury to return to full play						
	HR	95% CI	*p*-value	HR	95% CI	*p*-value
Characteristic	Unadjusted			Adjusted (n = 1158)		
Male	1.14	1.02–1.28	0.03	1.06	0.93–1.20	0.40
Class year						
Freshman				Ref	Ref	Ref
Sophomore				0.97	0.83–1.13	0.68
Junior				1.16	0.99–1.37	0.07
Senior				1.07	0.89–1.28	0.46
Fifth year				1.34	0.77–2.35	0.30
High-contact sport				1.01	0.87–1.18	0.86
Symptom count				0.98	0.97–0.99	< 0.001
Concussion history						
0				Ref	Ref	Ref
1				0.95	0.83–1.09	0.44
≥ 2				0.76	0.65–0.90	0.001
Academic return before symptom resolution*time to return academics				1.02	1.00–1.04	0.03
Academic return before symptom resolution				0.36	0.28–0.46	< 0.001
Time to return to academics				0.92	0.91–0.94	< 0.001

#### Return to Full Academics

The HR of full academic return for males compared to females was 1.22 (95% CI 1.07–1.38), and there was a 9% attenuation in the HR for sex (HR = 1.11, 95% CI 0.97–1.28) after adjustment for class year, high-contact sport, symptom count, concussion history, having academic accommodations, and greater time to symptom resolution (> median, 7 days) (Table 2). The greatest attenuation in the HR estimate for sex was due to having a longer time to symptom resolution (time to symptom resolution > median).

#### Return to Full Play

For males, the HR of return to full play was 1.14 (95% CI 1.02–1.28) compared to females, and there was a 7% attenuation in HR for sex (HR = 1.06, 95% CI 0.93–1.20) after adjustment for class year, high-contact sport, symptom count, concussion history, having academic return before symptoms resolved, and longer academic return (Table 2). The greatest attenuation in the HR estimate for sex was due to the interaction between having academic return before symptoms resolved and longer academic return.

#### Interaction by High/Low-Contact-Level

There was no evidence for interaction by high/low-contact sport level in the associations between sex and each of the three outcomes. Still, differences in the HR and 95% CI are apparent (Table 3), particularly for the outcome of return to full academics, and may be clinically significant.

**Table 3:** Hazard ratios for association between sex and time to recovery outcomes, stratified by high/low contact sport*

Outcome: Time from injury to symptom resolution												
Characteristic	HR	95% CI	*p*-value	HR	95% CI	p-value	HR	95% CI	*p*-value	HR	95% CI	*p*-value
	Unadjusted						Adjusted					
	Low contact			High contact			Low contact (n = 253)			High contact (n = 895)		
Male	1.29	0.96–1.72	0.90	1.16	1.01–1.33	0.04	1.24	0.91–1.69	0.16	1.12	0.97–1.29	0.12
Class year												
Freshman							Ref	Ref	Ref	Ref	Ref	Ref
Sophomore							0.76	0.55–1.06	0.10	0.93	0.78–1.11	0.44
Junior							1.07	0.76–1.51	0.71	1.08	0.89–1.30	0.45
Senior							0.88	0.58–1.35	0.56	1.06	0.87–1.30	0.55
Fifth year							0.87	0.21–3.55	0.84	0.86	0.47–1.58	0.64
Symptom count							0.95	0.93–0.97	< 0.001	0.93	0.92–0.95	< 0.001
Concussion history												
0							Ref	Ref	Ref	Ref	Ref	Ref
1							0.67	0.50–0.90	0.008	0.85	0.73–0.99	0.04
≥ 2							0.72	0.46–1.12	0.14	0.77	0.63–0.91	0.003

Outcome: Time from injury to return to full academics												
Characteristic	HR	95% CI	*p*-value	HR	95% CI	*p*-value	HR	95% CI	p-value	HR	95% CI	*p*-value
	Unadjusted						Adjusted					
	Low contact			High contact			Low contact (n = 234)			High contact (n = 804)		
Male	1.88	1.38–2.57	< 0.001	1.19	1.02–1.38	0.02	1.52	1.10–2.12	0.01	1.05	0.90–1.22	0.55
Class year												
Freshman							Ref	Ref	Ref	Ref	Ref	Ref
Sophomore							0.75	0.53–1.05	0.09	0.94	0.78–1.14	0.56
Junior							0.76	0.52–1.11	0.16	1.02	0.84–1.25	0.84
Senior							0.73	0.47–1.14	0.17	1.03	0.83–1.28	0.79
Fifth year							0.40	0.05–2.94	0.37	0.82	0.44–1.56	0.55
Symptom count							0.99	0.96–1.01	0.41	0.98	0.97–0.99	0.02
Concussion history												
0							Ref	Ref	Ref	Ref	Ref	Ref
1							1.08	0.79–1.49	0.62	0.99	0.84–1.17	0.93
≥ 2							0.77	0.47–1.26	0.30	0.88	0.73–1.06	0.18
Academic accommodations-yes							0.50	0.36–0.68	< 0.001	0.59	0.51–0.69	< 0.001
Symptom resolution > median							0.38	0.27–0.52	< 0.001	0.38	0.33–0.45	< 0.001

Outcome: Time from injury to return to full play												
Characteristic	HR	95% CI	*p*-value	HR	95% CI	*p*-value	HR	95% CI	*p*-value	HR	95% CI	*p*-value
	Unadjusted						Adjusted					
	Low contact			High contact			Low contact (n = 255)			High contact (n = 897)		
Male	1.32	0.99–1.76	0.06	1.11	0.97–1.28	0.14	1.08	0.79–1.47	0.64	1.04	0.91–1.21	0.51
Class year												
Freshman							Ref	Ref	Ref	Ref	Ref	Ref
Sophomore							0.97	0.70–1.36	0.87	0.94	0.79–1.13	0.52
Junior							1.33	0.94–1.88	0.11	1.12	0.93–1.35	0.23
Senior							1.00	0.67–1.52	0.98	1.10	0.90–1.35	0.35
Fifth year							1.08	0.26–4.45	0.92	1.40	0.76–2.58	0.29
Symptom count							0.97	0.95–0.99	0.02	0.98	0.96–0.99	0.001
Concussion history												
0							Ref	Ref	Ref	Ref	Ref	Ref
1							1.10	0.82–1.48	0.51	0.91	0.78–1.07	0.25
≥ 2							0.97	0.62–1.51	0.88	0.73	0.61–0.87	< 0.001
Academic return before symptom resolution*time to return academics							1.01	0.97–1.05	0.67	1.02	1.00–1.04	0.05
Academic return before symptom resolution							0.36	0.21–0.62	< 0.001	0.36	0.27–0.47	< 0.001
Time to return to academics							0.92	0.89–0.95	< 0.001	0.92	0.91–0.94	< 0.001

*n = 27 sports; High-contact: Basketball, Field hockey, Football, Ice hockey, Lacrosse, Rugby, Soccer, Sprint football, Water polo, Wrestling; Low-contact: Baseball, Cheerleading, Diving, Equestrian, Fencing, Golf, Gymnastics, Polo, Rowing/Crew, Sailing, Skiing, Softball, Squash, Swimming, Tennis, Track & field/Cross country, Volleyball

### Sensitivity Analyses

#### Role of School/Study Site

In models accounting for school/study site, the adjusted HR for sex was similar to our primary analysis for all three outcomes; symptom resolution, HR = 1.19 (95% CI 1.05–1.33); full academic return, HR = 1.08 (95% CI 0.94–1.25; p = 0.29); full play return, HR = 1.10 (95% CI 0.97–1.26) (Appendix Table 2).

#### Sports with Comparable Rules Among Men’s/Women’s Teams

Among athletes playing high-contact sports with comparable rules (basketball, soccer, rugby, water polo) only, the adjusted HR for sex was attenuated for outcomes symptom resolution (HR = 1.05, 95% CI 0.84–1.32) and full academic return HR = 1.01 (95% CI 0.79–1.30) compared to our primary analysis including all sports. The adjusted HR for sex was similar for outcome return to full play (HR = 1.12, 95% CI 0.97–1.26) (Appendix Table 3).

#### Missing Data

The characteristics among those with complete data compared those with missing data overall were similar. Those with complete data were slightly less likely to play a high-contact sport, although high-contact sports still represented the majority (n = 905, 78.0%). Those with complete data reported one fewer symptom (median = 11, 25th–75th percentiles: 7–15), and just more than half (n = 631, n = 54.4%) did not have a previous concussion (Appendix Table 1). In both sensitivity analyses, we examined the HR estimates for sex, and standard errors in our three fully adjusted models, and found them to be similar to the primary complete case analysis. As a result, we moved forward with the complete case cohort for our main analysis.

## Discussion

This cohort study focused on determining whether concussion recovery trajectories in the collegiate athlete population differ by sex, evaluating the role of baseline covariates, and accounting for the timing (length and sequence) of RTP outcomes. We found that cumulative, unadjusted time to symptom resolution, full academic return, and full play return was consistently lower among males compared to females, and that females lagged 1 day behind males in their recovery. When further stratified by high/low-contact sport, we found, on average, time to symptom resolution was the same among females, and longer for low-contact athletes among males. Time to academic return was on average longer for low-contact athletes among females, and shorter among males. Time to return to full play was on average longer for low-contact athletes among females and males. Aligning with these results, we found significant associations and a difference in hazard ratio by sex across outcomes, but these associations were attenuated after adjusting for available, a priori demographic and clinical characteristics.

This study was informed by previous, recent investigations of student-athletes with concussion in the Ivy-B1G study [8, 9]. Earlier work from 2013 to 2018 showed that roughly half of Ivy-B1G student-athletes had symptoms resolve ≤ 1 week, returned to academics ≤ 1 week, and returned to full athletic activity ≤ 2 weeks after injury [8]. Additional examination into the sequence of return to play stages revealed three distinct recovery profiles, where roughly one third (38.0%) of student-athletes had symptom resolution first, and then returned to full academics, and full sport, respectively (most consistent with the 2017 CISG consensus recommendations) [6]. In addition, roughly half (51.3%) of student-athletes returned to full academics, followed by symptom resolution, and then return to full sport [6] indicating that some athletes progress through the protocol stages in a different order. Given the nested nature of the RTP protocol [6, 7], these differences need to be accounted for in terms of determining cumulative RTP timelines, which this study adds. Specifically, we find that having longer time to symptom resolution, and also the sequence of having academic return before symptoms resolve and longer time to academic return, are major confounders in the relationship between sex and RTP timelines, shown by the largest attenuations in the HR for sex.

Some studies previously performed among collegiate athletes did not find statistically significant differences in time to symptom resolution, return to academics, and return to full play between males and females [8, 23, 24]. Our study uses novel methods and extends this work by examining recovery outcomes first in all men’s and women’s collegiate sports across participating schools, that is, beyond only men’s/women’s sports with comparable rules. We then account for level of contact in our models, as athletes playing high-contact or collision sports may be more likely to have sustained a concussion (and previous concussion(s)) in their sport given the higher incidence of concussion in these sports [8, 25]. In our analysis examining potential effect modification by sport contact-level, we stratified by high/low contact, and the interaction between sex and contact-level was not statistically significant for any of the three outcomes, but differences in the HR estimates for sex between high/low contact sports were apparent. These differences may be clinically significant, and warrant further study. In particular, our finding that a difference by sex is more apparent for low vs high-contact sports, especially for time to return to academics, may indicate a population and area of future focus in which to allocate more academic support and resources.

Another strength of our study was our ability to investigate recovery timelines among a large, homogeneous sample of division 1 collegiate student athletes from two athletic conferences, which has been a concern in terms of study quality for many previous studies with small sample sizes and/or where different participant characteristics, and also concussion definition and measurement approaches pose a challenge to interpreting results [7]. In our sensitivity analysis, accounting for school/study site, we found no apparent difference in the HR estimates across our models, and therefore, we concluded that for the purposes of this study, differences in recovery timelines are not attributed to management differences across participating schools. This is also encouraging, and suggests that Ivy-B1G schools may be operating according to national or international treatment and management guidelines for concussion from CISG and/or NATA, making the role of individual institutional differences less pertinent.

In our subgroup analysis among male/female high-contact-sport athletes with comparable rules, we found the HR estimates attenuated compared to our models among all sports (including football), and there were no statistically significant differences by sex in our adjusted models. Our results suggest that there may be something different about men’s or women’s team-only sports (e.g., football) compared to those who are included in the larger sample. Notably, though, this sample for this analysis represents roughly 25% of our SRC population, and so this analysis may be underpowered, and these results should be interpreted with caution. Still, this finding points to the need for robust future research that is focused on studying underlying features of men’s and women’s sports that may relate to concussion outcomes.

Taken together, this study contributes new evidence and further supports what is known about the role of sex and sport in recovery trajectories following sport-related concussion. Given the exploratory nature of our study, our findings also stimulate some considerations for investigating sex differences through targeted, future research in this population. Specifically, all student-athletes in the current study are members of institutions with robust athletic training programs, suggesting an overall enhanced concussion surveillance and management environment that may be explain some of the similarity in recovery outcomes among males and females, a phenomenon that has been found and described previously [23]. Persistent differences found here in recovery time by contact-level may indicate disparities across sports in other social/contextual factors, such as resource allocation, concussion surveillance and reporting, among others. Collegiate sport provides a unique context whereby student-athletes have been organized into groups (teams) based on biological sex. In determining differences in recovery between athletes, there may be underlying biological and physiological factors at play that combine with social and contextual factors to affect their experience after injury and the quality and timeline of their recovery. The ability to tease apart and identify opportunities to improve care and outcomes following concussion via areas/factors that are modifiable remains critical.

### Limitations

Our study makes important contributions to the literature on sex differences in SRC and recovery outcomes, specifically in terms methodological considerations when working with concussion surveillance data. Still, some limitations must be considered. First, our findings may not be generalizable to other collegiate athletic conferences and levels of play, however, this study extends research on patients who may present to emergency departments, primary care, and specialty clinics where diagnosis and close monitoring through progressed return to play may not be as feasible. Second, for 54% of student-athletes in our population, this was their first concussion ever documented. However, we are unable to know if some individuals in the study had multiple concussions during the study period (2015–2020), meaning their concussion characteristics and outcomes may be correlated, and this is a limitation. Still, those athletes with more than one concussion likely comprise a very small portion of the sample. Third, we were limited to considering symptom count (i.e., the total number of reported symptoms) instead of symptom severity, as symptom data from the Ivy-B1G Study are collected via a dichotomized check-list from the SCAT–22 symptom evaluation. This method of collecting symptoms is in the best interest of the Ivy-B1G study as a surveillance system, and we hope that our analysis here motivates future examinations of the role of symptom severity in recovery timelines. Of note, the nature of self-reported symptoms, and potential for inherent social desirability bias should be mentioned, as a common consideration for studies investigating reported symptoms after concussion. Data in the Ivy-B1G Study are collected by athletic trainers who work with student-athletes on each team, minimizing the threat of this in our study here. Fourth, immortal time bias can occur in observational studies when individuals in a cohort are followed while outcomes cannot occur. To minimize potential immortal time bias, whereby in observational studies, individuals in a cohort are followed while outcomes cannot occur, we approached our analysis carefully, given existing guidelines to the stepwise progression of our outcomes (time to symptom resolution, time to return to academics, and time to return to full play) as part of RTP protocols, and we accounted for this nesting of outcomes in our models. Finally, there was a notable amount of missing data, particularly in terms of RTP outcomes, however, we performed sensitivity analyses to investigate this, and our results were similar.

## Conclusion

Here, we found that among a cohort of collegiate athletes with sport-related concussion, time to sequential recovery outcomes appeared similar between male and female athletes, adjusting for observable confounders. Further differences in these outcomes between male and female athletes considering contact-level were evident, and may be important to consider clinically and should be examined in targeted, future research. Finally, we found evidence suggesting that differences between males and females may be largely attributed to and driven by differences in sports with a men’s or women’s team only, such as football. This should be explored further, and as it relates to future approaches to investigations of sex differences in recovery trajectories.

Overall, in this study, we present findings in the context of a large sample of collegiate athletes with concussion and we demonstrate practical considerations to using surveillance data in the field, which is a strength. This study makes contributions to understanding recovery timelines following concussion by use of robust methods and a thoughtful approach to examining missing outcome data, in addition to accounting for the nesting of RTP outcomes. Results from the present study will inform concussion management protocols at the collegiate level and the conversation around sex differences in recovery following concussion. Findings also inform future, planned qualitative work to further elucidate how collegiate athletes experience concussion recovery, considering differences among sport contact-level and athletes on men’s and women’s teams.

### Supplementary Information

Supplementary file.

## Data Availability

Data are not publicly available due to current data sharing policies of the Ivy League-Big Ten Epidemiology of Concussion Study.

## Abbreviations

SRC: Sport-related concussion
CISG: Concussion in Sport Group
NATA: National Athletic Training Association
RTP: Return-to-play
SCAT: Sport Concussion Assessment Tool
HR: Hazard ratio
CI: Confidence interval
