# Supplementary material for: Associations of Sex and Sport Contact-Level with Recovery Timelines Among Collegiate Athletes with Sport-Related Concussion
Source: Sports Med Open. 2024 Jul 29;10:86. doi: 10.1186/s40798-024-00742-4 (PMC11286888; doi:10.1186/s40798-024-00742-4)
Supplement: Supplementary file 1 — Supplementary file. [file 40798_2024_742_MOESM1_ESM.docx]

**SUPPLEMENTARY MATERIAL**

**Journal:** Sports Medicine Open

**Title:** Associations of sex and sport contact-level with recovery timelines among collegiate athletes with sport-related concussion

**Author list:**

Bernadette A. D’Alonzo PhD, MPH

Department of Biostatistics, Epidemiology and Informatics, Perelman School of Medicine, University of Pennsylvania, Philadelphia, PA, USA

Andrea L.C. Schneider MD, PhD

Department of Neurology, Perelman School of Medicine, University of Pennsylvania, Philadelphia, PA

Department of Biostatistics, Epidemiology and Informatics, Perelman School of Medicine, University of Pennsylvania, Philadelphia, PA, USA

Ian J. Barnett PhD

Department of Biostatistics, Epidemiology and Informatics, Perelman School of Medicine, University of Pennsylvania, Philadelphia, PA, USA

Christina L. Master MD

Department of Pediatrics, Perelman School of Medicine, University of Pennsylvania, Philadelphia, PA, USA

Center for Injury Research and Prevention, Children's Hospital of Philadelphia, Philadelphia, PA, USA

Abigail C. Bretzin PhD, ATC

Injury Prevention Center, Department of Emergency Medicine, University of Michigan, Ann Arbor, MI, USA

Douglas J. Wiebe PhD

Injury Prevention Center, Department of Emergency Medicine, University of Michigan, Ann Arbor, MI, USA

Ivy League-Big Ten Epidemiology of Concussion Study Investigators*

Ivy League-Big Ten Epidemiology of Concussion Study Investigators (listed alphabetically by institution): Beth Conroy, MS, ATC (Brown University); Thomas Bottiglieri, DO (Columbia University); Amy Sucheski-Drake, MD and Kathryn J. Harris, MS, ATC (Cornell University); Kristine A. Karlson, MD and Jonathan D. Lichtenstein, PsyD, MBA (Dartmouth College); Arun J. Ramappa, MD (Harvard University); Randy Ballard, MS, ATC (University of Illinois); Nicholas L. Port, PhD (Indiana University); Andrew R. Peterson, MD, MSPH (University of Iowa); Bradley D. Hatfield, PhD (University of Maryland); Mathew R. Saffarian, DO, (Michigan State University); James T. Eckner, MD (University of Michigan); Erin Moore, M.Ed, ATC and Suzanne Hecht, MD (University of Minnesota); Cary R. Savage, PhD and Kate Higgins, PsyD, ABPP-CN (University of Nebraska-Lincoln); Matthew J. Nerrie, MS, ATC (Northwestern University); Anthony Erz, MS, ATC and Brian J. Sennett, MD (University of Pennsylvania); Michael Gay, PhD, ATC (Pennsylvania State University); Sasha Steinlight, MD (Princeton University); Scott Lawrance, DHSc, ATC, MSPT (Purdue University); Jason Womack, MD and Carrie Esopenko, PhD (Rutgers University); Elizabeth C. Gardner, MD (Yale University).


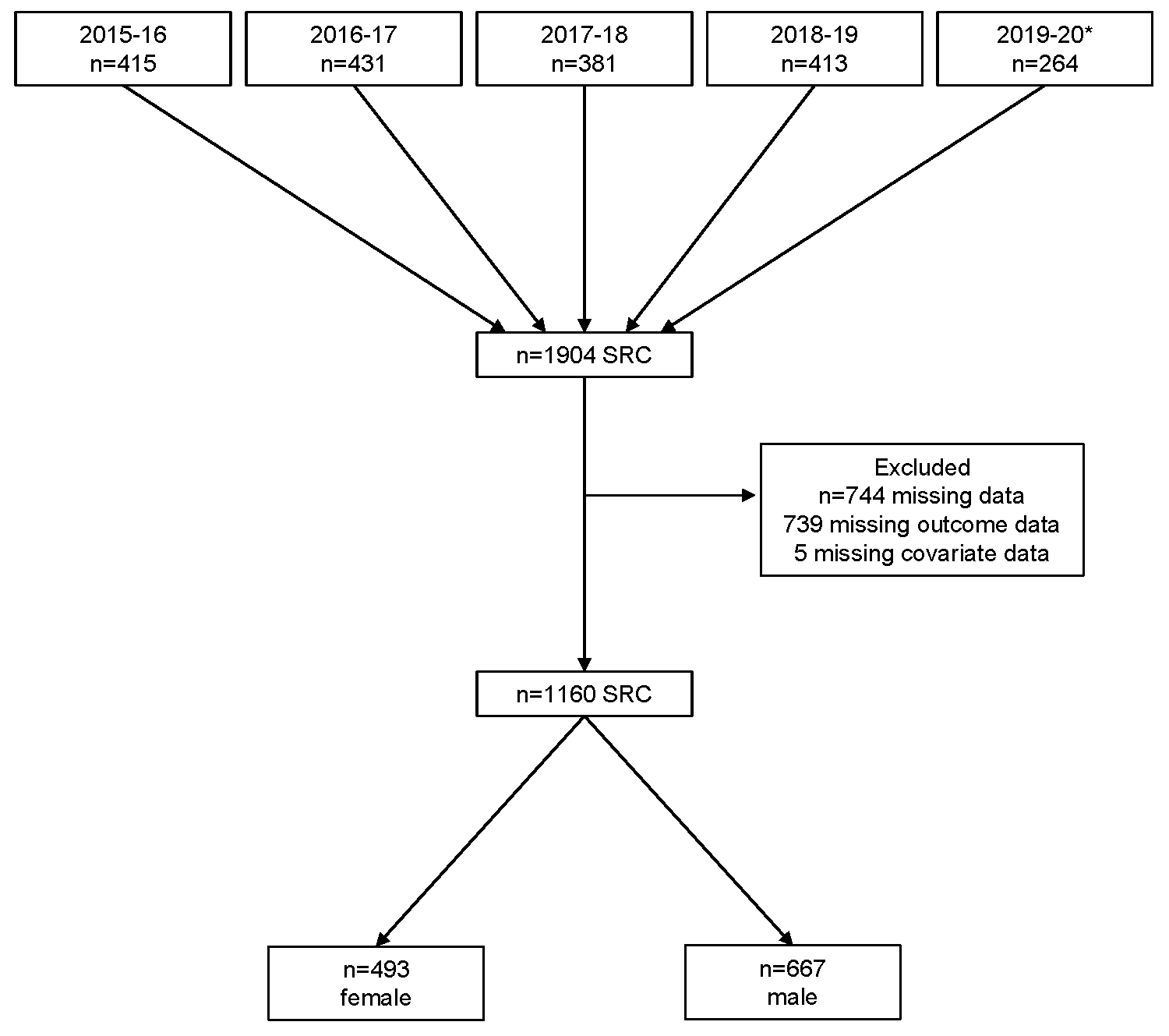


**Appendix Figure 1. CONSORT diagram depicting SRC cases included in analysis.**

*2019-20: Data collection through February 2020 due to COVID-19.

| **Appendix Table 1. Ivy-B1G SRC complete case cohort vs cases with missing values** | | | | |
| --- | --- | --- | --- | --- |
| Characteristic, n(%) | Overall | Complete case | Missing | p-value |
| Total | 1904 | 1160 (60.9) | 744 (39.1) |  |
| Male | 1125 (59.1) | 667 (57.5) | 458 (61.6) | 0.08 |
| Academic year |  |  |  |  |
| 2015-2016 | 415 (21.8) | 263 (22.7) | 152 (20.4) | 0.34 |
| 2016-2017 | 431 (22.6) | 246 (21.2) | 185 (24.9) |  |
| 2017-2018 | 381 (20.0) | 238 (20.5) | 143 (19.2) |  |
| 2018-2019 | 413 (21.7) | 256 (22.1) | 157 (21.1) |  |
| 2019-2020* | 264 (13.9) | 157 (13.5) | 107 (14.4) |  |
| Class year |  |  |  | 0.35 |
| Freshman | 505 (26.5) | 309 (26.6) | 196 (26.4) |  |
| Sophomore | 597 (31.4) | 353 (30.4) | 244 (32.9) |  |
| Junior | 466 (24.5) | 281 (24.2) | 185 (24.9) |  |
| Senior | 310 (16.3) | 204 (17.6) | 106 (14.3) |  |
| Fifth year | 24 (1.3) | 13 (1.1) | 11 (1.5) |  |
| Age, median (25^th^-75^th^ percentiles) | 20 (19-21) | 20 (19-21) | 20 (19-21) | 0.37 |
| High-contact sport | 1533 (80.5) | 905 (78.0) | 628 (84.4) | <0.001 |
| Symptom count, med (25^th^-75^th^ percentiles) | 11 (7-15) | 11 (7-15) | 12 (8-16) | <0.001 |
| Concussion history |  |  |  | 0.003 |
| 0 | 980 (51.5) | 631 (54.4) | 349 (46.9) |  |
| 1 | 543 (28.5) | 319(27.5) | 224 (30.1) |  |
| ≥2 | 381 (20.0) | 210 (18.1) | 171 (23.0) |  |
| Academic accommodations | 875 (48.9) | 578 (49.8) | 297 (47.1) | 0.28 |

| **Appendix Table 2. Adjusted hazard ratios for association between sex and time to recovery outcomes, accounting for school/site** | | | |
| --- | --- | --- | --- |
|  | HR | 95% CI | p-value |
| Outcome: Time to symptom resolution | | | |
| Characteristic | Adjusted (n=1148) | | |
| Male | 1.19 | 1.05-1.36 | 0.009 |
| Class year |  |  |  |
| Freshman | ref | ref | ref |
| Sophomore | 0.90 | 0.77-1.06 | 0.2 |
| Junior | 1.08 | 0.92-1.29 | 0.35 |
| Senior | 1.00 | 0.83-1.21 | 0.96 |
| Fifth year | 0.82 | 0.45-1.50 | 0.53 |
| High-contact sport | 1.08 | 0.93-1.27 | 0.31 |
| Symptom count | 0.93 | 0.92-0.94 | <0.001 |
| Concussion history |  |  |  |
| 0 | ref | ref | Ref |
| 1 | 0.82 | 0.71-0.94 | 0.005 |
| ≥2 | 0.80 | 0.68-0.95 | 0.01 |
| Outcome: Time to return to full academics | | | |
| Characteristic | Adjusted (n=1038) | | |
| Male | 1.08 | 0.94-1.25 | 0.29 |
| Class year |  |  |  |
| Freshman | ref | ref | ref |
| Sophomore | 0.92 | 0.78-1.10 | 0.36 |
| Junior | 0.97 | 0.81-1.16 | 0.73 |
| Senior | 0.99 | 0.81-1.22 | 0.95 |
| Fifth year | 0.75 | 0.38-1.45 | 0.39 |
| High-contact sport | 0.89 | 0.75-1.05 | 0.16 |
| Symptom count | 0.98 | 0.97-0.99 | 0.008 |
| Concussion history |  |  |  |
| 0 | ref | ref | ref |
| 1 | 1.06 | 0.91-1.23 | 0.48 |
| ≥2 | 0.89 | 0.75-1.07 | 0.22 |
| Academic accommodations-yes | 0.57 | 0.49-0.66 | <0.001 |
| Symptom resolution>median | 0.34 | 0.29-0.40 | <0.001 |
| Outcome: Time to return to full play | | | |
| Characteristic | Adjusted (n=1443) | | |
| Male | 1.1 | 0.97-1.26 | 0.14 |
| Class year |  |  |  |
| Freshman | ref | ref | ref |
| Sophomore | 0.97 | 0.82-1.13 | 0.62 |
| Junior | 1.23 | 1.04-1.46 | 0.02 |
| Senior | 1.11 | 0.92-1.34 | 0.27 |
| Fifth year | 0.88 | 0.49-1.61 | 0.7 |
| High-contact sport | 1.02 | 0.87-1.20 | 0.78 |
| Symptom count | 0.96 | 0.95-0.98 | <0.001 |
| Concussion history |  |  |  |
| 0 | ref | ref | ref |
| 1 | 0.95 | 0.82-1.09 | 0.46 |
| ≥2 | 0.78 | 0.65-0.92 | 0.004 |
| Academic return before symptom*time to return academics | 1.01 | 0.99-1.03 | 0.47 |
| Academic return before symptom | 0.38 | 0.29-0.50 | <0.001 |
| Time to return to academics | 0.91 | 0.90-0.93 | <0.001 |

| **Appendix Table 3. Adjusted hazard ratios for association between sex and time to recovery outcomes, among athletes from men’s/women’s high-contact sports with comparable rules (n=325)*** | | | |  |
| --- | --- | --- | --- | --- |
|  | HR | 95% CI | p-value | |
| Outcome: Time to symptom resolution | | | |  |
| Characteristic | Adjusted (n=323) | | | |
| Male | 1.05 | 0.84-1.32 | 0.66 | |
| Class year |  |  |  | |
| Freshman | ref | ref | ref | |
| Sophomore | 0.90 | 0.76-1.35 | 0.92 | |
| Junior | 1.08 | 0.85-1.60 | 0.33 | |
| Senior | 1.04 | 0.52-1.02 | 0.07 | |
| Fifth year | 0.85 | 0.09-1.61 | 0.19 | |
| Symptom count | 0.94 | 0.92-0.96 | <0.001 | |
| Concussion history |  |  |  | |
| 0 | ref | ref | ref | |
| 1 | 0.96 | 0.74-1.23 | 0.73 | |
| ≥2 | 0.86 | 0.63-1.18 | 0.35 | |
| Outcome: Time to return to full academics | | | |  |
| Characteristic | Adjusted (n=270) | | | |
| Male | 1.01 | 0.79-1.30 | 0.93 | |
| Class year |  |  |  | |
| Freshman | ref | ref | ref | |
| Sophomore | 0.95 | 0.70-1.30 | 0.75 | |
| Junior | 1.05 | 0.74-1.48 | 0.78 | |
| Senior | 0.83 | 0.57-1.21 | 0.33 | |
| Fifth year | 0.41 | 0.06-3.04 | 0.39 | |
| Symptom count | 0.96 | 0.93-0.98 | 0.001 | |
| Concussion history |  |  |  | |
| 0 | ref | ref | ref | |
| 1 | 1.02 | 0.78-1.35 | 0.86 | |
| ≥2 | 0.79 | 0.56-1.12 | 0.20 | |
| Academic accommodations-yes | 0.67 | 0.51-0.86 | 0.002 | |
| Symptom resolution>median | 0.41 | 0.31-0.54 | <0.001 | |
| Outcome: Time to return to full play | | | |  |
| Characteristic | Adjusted (n=324) | | | |
| Male | 1.12 | 0.89-1.40 | 0.35 | |
| Class year |  |  |  | |
| Freshman | ref | ref | ref | |
| Sophomore | 1.17 | 0.88-1.57 | 0.29 | |
| Junior | 1.52 | 1.10-2.09 | 0.01 | |
| Senior | 1.17 | 0.84-1.64 | 0.35 | |
| Fifth year | 1.57 | 0.35-7.03 | 0.55 | |
| Symptom count | 0.97 | 0.94-0.99 | 0.004 | |
| Concussion history |  |  |  | |
| 0 | ref | ref |  | |
| 1 | 0.77 | 0.59-0.99 | 0.05 | |
| ≥2 | 0.59 | 0.42-0.82 | 0.002 | |
| Academic return before symptom*time to return academics | 0.99 | 0.95-1.02 | 0.47 | |
| Academic return before symptom | 0.58 | 0.37-0.92 | 0.02 | |
| Time to return to academics | 0.94 | 0.92-0.97 | <0.001 | |
| *Men’s/Women’s contact sports with comparable rules: Basketball, Soccer, Rugby, Water polo (all high-contact sports, contact-level variable omitted) | | | |  |
